# Diagnosis and Pattern Identification of Intrathoracic Malignant Melanoma Metastasis: A Retrospective Single Center Analysis

**DOI:** 10.3390/diagnostics12092254

**Published:** 2022-09-18

**Authors:** Matteo Fontana, Laura Rossi, Federica Ghinassi, Roberto Piro, Chiara Scelfo, Sofia Taddei, Eleonora Casalini, Patrizia Ruggiero, Chiara Pollorsi, Bianca Beghe’, Caterina Longo, Nicola Facciolongo

**Affiliations:** 1 Pulmonology Unit, Department of Medical Specialties, Azienda Unità Sanitaria Locale—IRCCS of Reggio Emilia, Viale Risorgimento 80, 42123 Reggio Emilia, Italy; 2 Respiratory Diseases Unit, Department of Medical, Surgical, Maternal-Infantile and Adult Sciences, Azienda Ospedaliero-Universitaria of Modena “Policlinico”, University of Modena and Reggio Emilia, Largo del Pozzo 71, 41124 Modena, Italy; 3 Department of Surgery, Medicine, Dentistry and Morphological Sciences with Interest in Transplantation, Oncology and Regenerative Medicine, Faculty of Medicine and Surgery, University of Modena and Reggio Emilia, Largo del Pozzo 71, 41124 Modena, Italy; 4 Skin Cancer Center, Department of Medical Specialties, Azienda Unità Sanitaria Locale—IRCCS of Reggio Emilia, Viale Risorgimento 80, 42123 Reggio Emilia, Italy

**Keywords:** melanoma, neoplasm metastasis, bronchoscopy, thoracoscopy

## Abstract

The lung is a frequent site of secondary malignancies. Melanoma is a malignant tumor originating from melanocytes, that accounts for the majority of death related to skin cancers. In advanced stages, it can also present with intrathoracic metastasis, particularly in the lungs, but infrequent intrathoracic manifestations are possible. A retrospective analysis of the cases referred to the pulmonary endoscopy unit of the hospital of Reggio Emilia in the last 10 years (since December 2012) was carried out, discovering 17 cases of melanoma metastasis with thoracic localizations, either with or without a diagnosis of primary melanoma. Four repetitive patterns of clinical-radiological presentation have been identified and described through the same number of paradigmatic clinical cases: nodal involvement (35%), lung mass(es) (41%), diffuse pulmonary involvement (12%), and pleural involvement (12%). These different presentations imply the use of different diagnostic techniques, with an overall high diagnostic yield (87.5%). Finally, a brief analysis of survival based on the pattern of presentation has been performed, finding no statistically significant differences between the four groups at metastasis diagnosis (*p*-value = 0.06, median survival of respectively 54, 8, 9, and 26 months from metastasis diagnosis), while there is a significant difference considering patients with lung involvement versus nodal/pleural involvement (*p* = 0.01).

## 1. Introduction

Malignant neoplasms, in their advanced stages, frequently involve lung and mediastinal lymph nodes [1]. When lungs are involved, the prognosis is often scarce but it depends on the site of the primary tumor [2,3]. Malignant melanoma is a neoplasm accounting for the great majority of skin cancer deaths [4]; its tendency to give distant metastases is well-known, and the lung is an elective site of melanoma metastases [5], also after a considerable amount of time [6]. Malignant melanoma could also derive from melanocytes present within the uvea: in this case, the biological behavior of the neoplasm is different and the liver is the primary site of metastasis, although intrathoracic secondarisms are also possible [7,8,9]. Nevertheless, the lung is not the only intrathoracic organ involved in melanoma metastasis: pleura [10,11] and intrathoracic lymph-nodes [12] can be other sites of thoracic metastasis from melanoma, as well as the sole endobronchial presentation [13,14]. Black bronchoscopy is an expression conceived in 2003 by Packham and colleagues [15] in order to describe the pathognomonic aspect of an endobronchial mass secondary to malignant melanoma. Literature data reported different patterns of metastasis to find out the prognostic impact of these different thoracic involvements [16,17]. In recent years, imaging tools and in particular interventional pulmonology diagnostic techniques have grown exponentially [18], giving us the possibility to promptly identify metastatic diseases and define their real extension in the thorax, in order to give the patient the best treatment, even the surgical resection. It is documented that surgical resection of intrathoracic metastasis is associated with a better prognosis, obviously depending on the number of metastasis and the completeness of surgery [19]. Thus, the role of an interventional pulmonologist is to give a diagnosis with the highest possible confidence and in the shortest period. The aim of this study is to evaluate the patterns of presentation of intrathoracic metastatic melanoma (MM), to analyze the diagnostic techniques adopted in order to reach the diagnosis and their diagnostic yield with a particular focus on those that allow us a direct visualization of the pathology, and eventually try to investigate the prognostic impact of different site(s) of intrathoracic metastasis.

## 2. Materials and Methods

### 2.1. Patients

This is a retrospective monocentric study focusing on metastatic melanoma patients referring to the interventional pulmonology unit from December 2012 to July 2022 at the center of Reggio Emilia for histological confirmation. We reviewed the informatic and paper databases of the unit and identified all the patients and, within them, some exemplary cases. After identifying the paradigmatic cases with a typical radiological and clinical presentation, we tried to cluster all the collected patients into cohorts, in order to observe an eventual repetitive scheme. For each pattern of presentation, we have checked the diagnostic yield of the procedure adopted in every specific case. Furthermore, anagraphics and data linked to the diagnosis of malignancy have been collected and divided between the groups. Finally, a secondary analysis has been performed sorting the patients into two greater cohorts, the ones with primary lung involvement and the others with nodal and pleural/parietal involvement.

### 2.2. Diagnostic Techniques

Focusing on the techniques adopted for the diagnosis of intrathoracic melanoma, first of all, radiological imaging and nuclear medicine tools have been adopted in order to evaluate the presence of this metastasis. Chest X-ray was the first level diagnostic tool adopted, with an Optimus 80 X-ray system, Philips Healthcare, used for this purpose. As a second-level exam, CT (computed tomography) scans were performed using a 128-slice scanner (Somatom Definition Edge, Siemens Healthineers, Erlangen, Germany) before and after contrast media injection, with the patient in the supine position, during end-inspiration. Scanning parameters were: tube voltage 120 KV, automatic tube current modulation, collimation width 1.25 mm, acquisition slice thickness 2.5 mm, and interval 1.25 mm. Magnetic resonance imaging (MRI) scans were obtained using a 1.5 T MR scanner (Philips Ingenia, Philips Healthcare, Andover, MA, USA) with a scanning protocol including respiratory triggered T2-weighted sequences with and without fat signal suppression, and pre- and post-contrast fat-suppressed T1-weighted gradient-echo sequences. Finally, all PET (positron emission tomography)-CT examinations were acquired by using a hybrid PET/CT system (Discovery STE, GE Healthcare, Chicago, IL, USA) with a 3.30 min emission scan/bed and CT-attenuation correction. Patients were required to fast for at least 4 h before intravenous injection of ^18^F-FDG (fluorodeoxyglucose, 3.7 MBq/kg). A low-dose, non-contrast-enhanced CT scan was carried out for PET co-registration. A whole-body emission scan was performed 60 min after ^18^F-FDG injection from the base of the skull to the proximal femora. All interventional pulmonology procedures have been performed in our endoscopy suite, with the use of an Olympus videobronchoscope (BF-190) or videoechobronchoscope (UC-180F); we used during endoscopic procedures traditional TBNA (transbronchial needle aspiration) needles (Olympus Smoothshot NA 401D-1521, size 21 Gauge), EBUS (endobronchial ultrasound) needles (Olympus Vizishot NA-2015K-4022, size 22 Gauge) and biopsy forceps (Olympus Endojaw FB-231D, Olympus Medical, Tokyo, Japan). Furthermore, we used HS Hospital Service Chiba needles EC 2115, size 21 Gauge, for the transthoracic needle aspiration (TTNA) under fluoroscopy guidance with a Philips BV Endura9 DO-618 C-arm present within the endoscopy suite and used also for the transbronchial biopsies. Finally, we used Richard Wolf PANOVIEW telescopes, size 4 mm, 0°–30°, and rigid optic biopsy forceps size 7 mm for the medical thoracoscopies. All the diagnostic techniques and instruments are summarized in Table 1.

### 2.3. Statistical Analysis

The data are presented with descriptive statistics; the comparisons between two groups of continuous variables have been performed with a *t*-test with Welch correction or with the Mann–Whitney test where appropriate while comparisons between more than two groups of continuous variables have been performed with Brown–Forsythe and Welch ANOVA test and Kruskal–Wallis test, where appropriate. For categorical variables, the chi-square test and Fisher’s exact test have been applied, where appropriate. Finally, survival analysis was carried out: the data on survival from the first diagnosis of melanoma and from the diagnosis of metastasis have been collected, analyzed with the log-rank test, and pictured out with Kaplan-Meier surviving curves. GraphPad Prism version 8.2 for MacOS (GraphPad Software, San Diego, CA, USA, www.graphpad.com (accessed on 31 July 2022)) is the software we used for the statistical analysis. This study has been approved by the Institutional Review Board of the Azienda USL—IRCCS Santa Maria Nuova of Reggio Emilia.

## 3. Results

17 cases of intrathoracic metastatic melanoma have been collected. All those patients had a history of resected melanoma in various cutaneous sites, except for one of them with a previous uveal melanoma and two of them in which the primary site was not known. Four patterns of presentation have been identified: (1) lymph nodal involvement, defined as enlargement of mediastinal or hilar lymph nodes with a minimum diameter >1 cm; (2) solitary lung mass or multiple masses, defined as the presence of one or more pulmonary lesions with a diameter ≥3 cm; (3) diffuse parenchymal infiltration of the lung, defined as the presence of multiple lesions involving both lungs and at least one lobe in each lung, consisting of nodules with a random distribution (i.e., typical of metastasis) or carcinomatous lymphangitis; (4) pleural/parietal involvement, defined as the presence of lesions involving the pleura and/or the chest wall. Within these groups, a patient for each of them has been chosen due to the paradigmatic clinical, radiological and diagnostic course, right away presented and synthetized in Table 2.

### 3.1. Case 1

**Figure 1:**
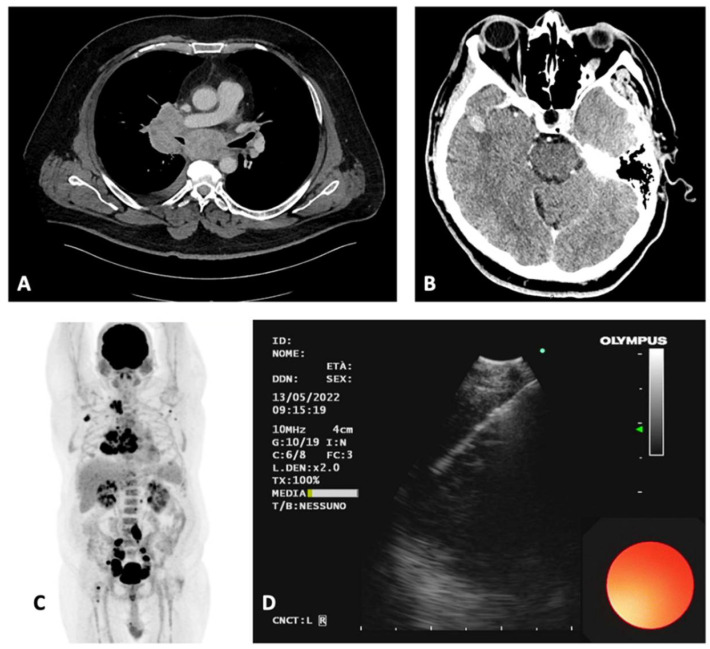
Case 1. (**A**) Axial contrast-enhanced CT scan showing multiple mediastinal adenopathies. (**B**) encephalic CT showing a right temporal lobe metastasis. (**C**) FDG-PET scan showing uptake of the mediastinal and abdominal lymphoadenopathies. (**D**), EBUS-TBNA of the subcarinal lymph node, station 7 according to IASLC. CT, computerized tomography; FDG- PET, fluorodeoxyglucose positron emission tomography; EBUS-TBNA, endobronchial ultrasound with transbronchial needle aspiration; IASLC, International Association for the Study of Lung Cancer.

A 53-year-old man with a history of smoking presented to the emergency department because of a two months history of fatigue, low-grade fever, weight loss, and cough. Their past medical history showed a surgical excision of melanoma on his left leg approximately 7 years before, arterial hypertension, obesity, and anxiety. The chest X-ray showed mediastinal enlargement, a contrast-enhanced total body CT scan showed two brain metastases (located in the right temporal lobe and in the left parietal lobe), bulky lymph nodes enlargement in the subcarinal and paratracheal stations, as well as in the retroperitoneal space and in the iliac chain bilaterally. He was admitted to the pulmonology unit where an FDG-PET scan was performed and then a bronchoscopy with EBUS-TBNA on the subcarinal station (due to the dimensions, radiological, and echographic features) has been carried out, showing the presence of a nodal metastasis of melanoma (Figure 1). BRAF tested positive for the V600E mutation and a first-line therapy with dabrafenib and trametinib was started. The disease remained stable after the initiation of treatment, with a mild tendency to the reduction of the thoracic, abdominal, and encephalic manifestations.

### 3.2. Case 2

**Figure 2:**
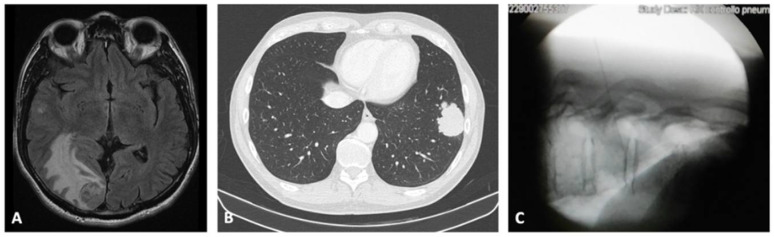
Case 2. (**A**) Axial T2WI/FLAIR MRI of the major brain lesion in the right occipital lobe. (**B**) axial CT scan of the rounded lung mass of the left lower lobe. (**C**) transthoracic needle aspiration with fluoroscopy guidance. MRI, magnetic resonance imaging; CT, computerized tomography.

A 60-year-old nonsmoker man presented to the oncology unit for the presence of multiple brain masses observed on MRI, that has been performed because of the onset of dysarthria and involuntary left eye movement. He was diagnosed with a malignant melanoma of the back 12 years before and treated with a surgical resection. The sentinel lymph node was negative (stage IIC following the American Joint Committee in Cancer staging). The CT scan performed for the staging of the disease showed a 5 cm left lung mass in the lower lobe, which has been sampled with a transthoracic fluoroscopy-guided needle aspiration with an 18 Gauge needle, reaching the diagnosis of metastatic malignant melanoma (Figure 2). In this case, the transthoracic maneuver could guarantee good access for the diagnosis and in particular, it was a less invasive technique than the biopsy of the encephalic tissue. BRAF tested positive for the V600E mutation and the patient started the therapy with dabrafenib and trametinib. After initial clinical improvement, the patient developed a progression of the disease and died 9 months after the diagnosis of lung metastasis.

### 3.3. Case 3

**Figure 3:**
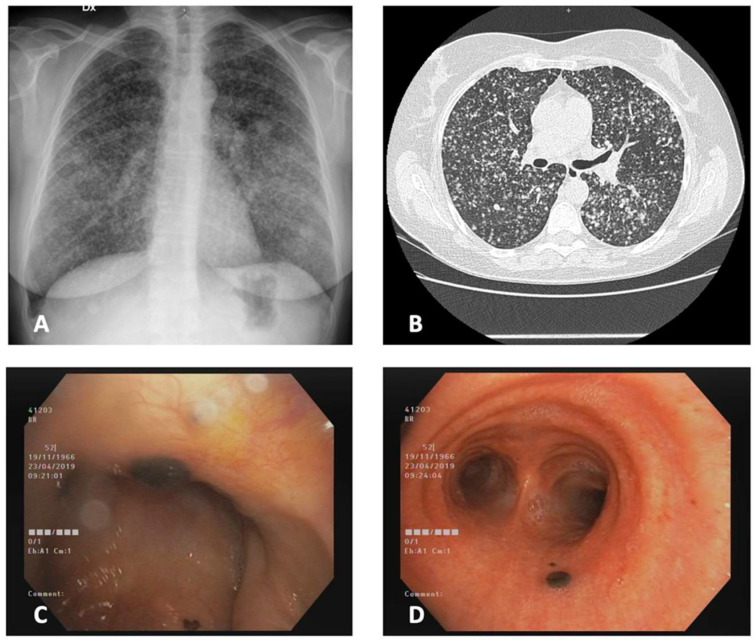
Case 3. (**A**) Antero-posterior chest X-ray at presentation showing a diffuse reticulonodular alteration. (**B**) CT scan at presentation, showing multiple micronodules with a random distribution, suggestive of metastasis. (**C**,**D**) nasal and tracheobronchial black plaques, consistent with metastasis. CT, computerized tomography.

A 52-year-old woman, a heavy smoker, with a past medical history significant for surgical resection of melanoma of the scalp approximately 11 years before the presentation (with a relapse, also treated with surgical resection), has been sent to the pulmonology unit after the execution of chest X-rays showing diffuse bilateral micronodular thickening of the lungs. Her symptoms were dyspnea and weight loss. A CT scan showed a high number of nodular and micronodular opacities, with a random distribution, in both lungs. She was admitted to the pulmonology unit and a bronchoscopy with bronchoalveolar lavage (BAL) and transbronchial biopsy were performed. At endoscopic inspection, endonasal, endotracheal, and endobronchial black plaques were visible (Figure 3); both BAL and biopsy highlighted the presence of melanoma metastasis. The bronchoscopy with transbronchial biopsy was chosen due to the high diagnostic yield with the micronodular distribution of opacities. The biomolecular analysis showed the presence of V600E mutation of BRAF. The staging showed also secondary localizations at the bones, liver, spleen, kidney, and lymph nodes. The patient started the therapy with dabrafenib and trametinib but after a new progression, she died 4 months after the endoscopic diagnosis.

### 3.4. Case 4

**Figure 4:**
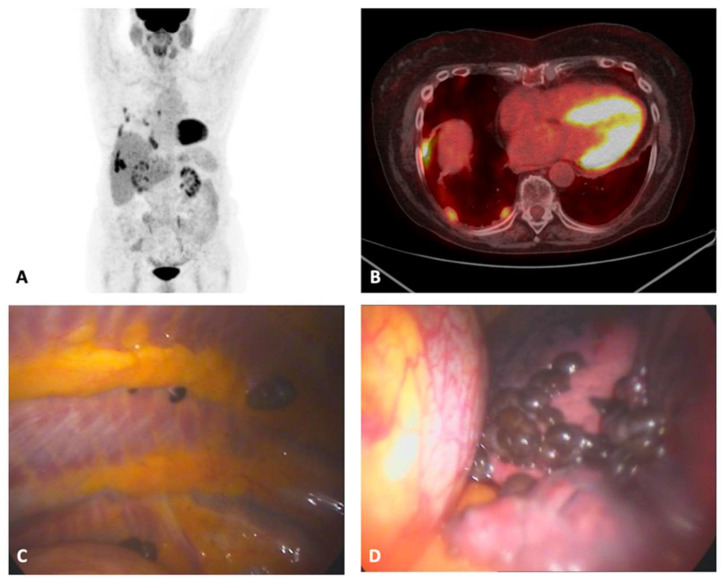
Case 4. (**A**,**B**) FDG-PET scans at presentation showing multiple right pleural thickenings with FDG uptake. (**C**,**D**) thoracoscopic view of the metastatic pleural plaques. FDG-PET, fluorodeoxyglucose positron emission tomography.

An 82-year-old woman with no history of smoking, presented to the pulmonology unit due to mild dyspnea and chest pain on the right side. Her past medical history showed a gastric ulcer, a past pleuritis in her young years, and dyslipidemia. She performed a CT scan highlighting multiple pleural nodulations, together with a mild pleural effusion, and subsequently an FDG- PET was performed showing an abnormal FDG uptake. She was scheduled for the execution of a medical thoracoscopy. This exam showed the presence of multiple black nodulations on the parietal and visceral pleura (Figure 4). Multiple biopsies were performed showing a spindle cell melanoma, with unknown primary origin. The thoracoscopic access was in this case the only one that can reach a confident diagnosis given the clinical presentation, considering the relatively low diagnostic yield and the technical difficulty in performing a closed pleural biopsy. Molecular biology did not show any mutation. Immunotherapy with nivolumab was started and modified the year after due to a disease progression with paclitaxel. The pathology exhibited a slow progression during the following two years but the patent did not report any new symptoms though the therapy was continued. She died approximately three years after the diagnosis of pleural metastases.

Of the total patients, six (35%) presented with a predominant lymph node involvement, with or without other extra thoracic site of metastasis, seven (41%) presented with one or more lung masses, two patients (12%) presented with a diffuse lung parenchymal involvement, with a predominant nodular pattern, while two patients (12%) presented with a primary pleural or thoracic wall involvement. In these patients, different diagnostic techniques have been applied. For the first group of patients, traditional TBNA (transbronchial needle aspiration) or EBUS-TBNA have been used with a diagnostic yield of 100%; for the second group, in two cases fluoroscopy-guided TTNA has been used and resulted in a definitive diagnosis, in one case bronchial biopsy (BB) reached the diagnosis, in one case traditional TBNA was diagnostic, in one case the bronchoscopy was not performed with a diagnostic aim and in the other two cases transbronchial biopsy and EBUS-TBNA did not reach the definitive diagnosis (overall four of six diagnoses reached with a diagnostic yield of 66%); in the third group, transbronchial lung biopsy (TBB), BAL or simple bronchial biopsies gave the diagnosis in two cases (100%); in the fourth and last group of presentation, medical thoracoscopy (MT) and pleural biopsy (PB) have been used as elective diagnostic procedures reaching the final diagnosis in both cases (diagnostic yield of 100%). Taken together, all the procedures for the whole cohort of patients had a diagnostic yield of 87.5% for the diagnosis of intrathoracic metastatic melanoma.

After collecting the anagraphics and clinical data of the patients and sorting them between the four groups, we did not observe a statistically significant difference in the age of the first presentation of the primary neoplasms (*p* = 0.08), although it was higher for the pleural involvement. Furthermore, a statistically significant difference was not observed in the years between the first presentation and lung metastasis, though it was higher for the patients with a parenchymal diffuse infiltration (*p* = 0.06). No statistically significant differences were detected between groups in age at metastasis, number of other metastasis sites, sex, Breslow thickness, BRAF mutation, and diagnostic yield of the interventional pulmonology procedure (see Table 3).

The survival analysis did not show any statistically significant difference between all the groups and between every single group with each other, although there is a tendency for a worse prognosis at the diagnosis of intrathoracic metastasis for the diffuse parenchymal pattern, as compared with the others. The median survival, after the diagnosis of melanoma and after the diagnosis of thoracic metastasis in months was respectively 130 and 54 months for the patients with lymph node involvement, 76 and 8 months for the patients with lung mass(es), 262 and 9 months for the patients with diffuse parenchymal involvement and finally 31.5 and 26 months for those with pleural involvement (*p*-values respectively of 0.06 and 0.08). The total median survival after the diagnosis of melanoma was 76 months while after the diagnosis of thoracic metastases was 11 months.

**Figure 5:**
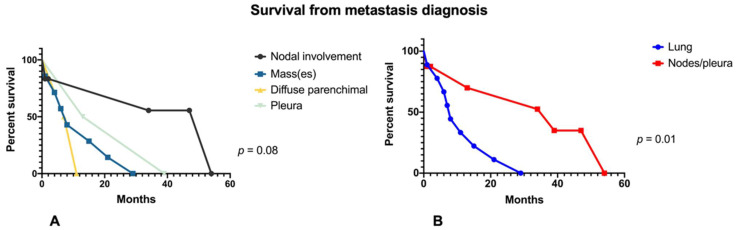
Kaplan-Meier survival curve of different patterns of lung metastasis. (**A**) comparison of the four identified patterns; (**B**) comparison of patients with lung involvement versus patients with nodal and pleural/parietal involvement. *p* is measured with the log-rank test.

We have also sorted the cases dividing them into two bigger cohorts: those with a dominant lung parenchymal involvement (i.e., with a diffuse involvement and the presence of masses) and those with a parenchymal sparing (i.e., nodal involvement and parietal/pleural involvement). All the previous features are showed for those groups in Table 4. In this case, we analyzed all the previous features finding only a significant difference between the groups in the survival after the metastasis diagnosis, significantly lower for the first group (8 versus 39 months with a *p* = 0.01), as shown in Figure 5.

## 4. Discussion

In this retrospective study, we identified a repetitive pattern of intrathoracic metastasis of MM, searching in the interventional pulmonology database of our center and starting from the description of four paradigmatic clinical cases. Our main interest was to find out the diagnostic techniques used for the acknowledgment of the pathology and subsequently identify the diagnostic yield of these techniques. In particular, the overall high diagnostic yield (87.5%) highlights that endoscopic techniques are a valuable instrument for the diagnosis of intrathoracic MM. Starting from bronchoscopy, two are the main tools that arose in the last years for early and precise diagnostic purposes: EBUS-TBNA and TBB. More simple endoscopic techniques are the traditional (or blind) TBNA and the BB: the first one is at this point less and less utilized due to the abundant amount of material and preciseness that EBUS-TBNA can guarantee [20], while the second is applicable only in cases where there is a clear endobronchial invasion. EBUS-TBNA is characterized by a high diagnostic accuracy on mediastinal lymphadenopathies, even for patients with extrathoracic malignancies [21]. Furthermore, it can reach many different thoracic nodal stations compared with other diagnostic techniques, also with the concomitant use of the echobronchoscope through the esophagus [22]. On the other hand, when the pathology involves primarily the lung parenchyma, the endoscopic tool we have is represented by the TBB with different guide systems and instruments (fluoroscopy, radial EBUS, thin and ultrathin scopes, lung navigation systems): this specific technique has evolved through years and has reached higher accuracy also for more peripheral and smaller lung lesion, with an excellent safety profile [23,24,25]. Focusing on the other procedures used, MT is another established endoscopic technique with a high diagnostic yield and an excellent safety profile [26], especially if compared with other diagnostic techniques [27]. On the other hand, non-endoscopic techniques are also valuable tools for the diagnosis of intrathoracic diseases: for example, fluoroscopy-guided TTNA is a simple technique useful for diagnosing large thoracic masses, with a lower radiation exposure compared with CT-guided techniques [28,29]. Finally, a closed pleural biopsy is another easy and simple technique used by interventional pulmonologists [30], however, characterized by a lower diagnostic yield compared with other procedures [27]. The diagnostic yield of the procedures utilized is an overall 87.5%, concordant with literature data; another quality of these techniques, in particular, the endoscopic ones and especially in melanoma metastasis, is the possibility to orient the diagnosis after the visualization of the pathological endoscopic picture, which in some cases present peculiar findings [31]. It is worth analyzing the two missed diagnoses: in the first case, the transbronchial biopsy did not reach the peripheral pulmonary lesion of approximately 3 cm, visualized also with the radial EBUS probe, probably because of the eccentric location from bronchi; in the second case, a patient with a known history of sarcoidosis, presenting with incremental uptake of FDG on those nodes and the appearance of lung nodules, EBUS-TBNA was performed on the nodes and the nodules have not been investigated, only confirming the presence of granulomas in lymph nodes. Alternative diagnostic techniques could have been fluoroscopy-guided TTNA for the first case and TBB for the second case.

Based on our data, we demonstrated that there are no significant differences in patients’ features between different patterns, in terms of survival, anagraphics, and pathological features. A statistically significant difference has been found in survival from metastasis presentation dividing patients between those with primary lung involvement (i.e., with diffuse parenchymal infiltration and lung masses) and those with a dominant nodal involvement or pleural/parietal involvement: this is concordant with literature data highlighting also the correlation between survival and the number of lung nodules [5,32]. This is probably due to the tendency of the pathology, in the first group, to have a hematogenous spreading and consequently to be a more diffuse and aggressive disease, therefore more difficult to monitor and promptly treat.

This study has some limitations. First, the limited number of cases hampers the survival analysis and the comparison between the four patterns of presentation. Second, the retrospective nature of the study and the monocentric site. Finally, the use of a diagnostic technique in these patients has not been compared directly with one another but chosen based on the presentation and the expertise of the interventional pulmonologist in that particular moment, making it difficult to deem one superior to another for a specific presentation.

On the other hand, we believe that this work focuses on the diagnosis of intrathoracic MM and offers useful practical clues in real life in particular regarding the diagnostic approach, and that it gives also prognostic information, following those already presented in the literature. Indeed, MM has a poor prognosis and, although nowadays there are growing pieces of evidence of efficacious treatment against this pathology [33,34], there is still a need to better understand the underlying mechanisms of the development of lung and other intrathoracic metastasis [35,36], in order to better intercept the predisposed patients and to quickly diagnose them with the proper technique.

## 5. Conclusions

This retrospective study on intrathoracic metastatic melanoma confirms that interventional pulmonology techniques can provide a reliable diagnostic tool for this pathology. The presence of repetitive patterns of intrathoracic metastasis has been postulated, implying a different diagnostic approach. This evidence could be useful for professionals managing patients with a previous diagnosis of melanoma and suspected thoracic repetition, in order to reach with the highest possible confidence a histologic diagnosis. Further research is needed regarding the tendency of melanoma to give different patterns of thoracic involvement, so as to predict and intercept the development of a particular pattern of metastasis, in order to provide a fast diagnosis and a personalized treatment.

## Figures and Tables

**Table 1:** Diagnostic techniques adopted and features of the correspondent instruments.

Diagnostic Technique	Instruments and Specifics
X-ray	Optimus 80 X-ray system, Philips Healthcare
Contrast-enhanced CT-scan	Somatom Definition Edge, Siemens Healthineers, 128-slice scanner
Contrast-enhanced MRI	Philips Ingenia, Philips Healthcare, 1.5 T MR scanner
FDG-PET	Discovery STE, GE Healthcare, PET/CT system with 3.30 min emission scan/bed and CT-attenuation correction
Videobronchoscope	Olympus BFH-190
EBUS	Olympus UC 180F
C-arm	Philips BV Endura9 DO-618
TBNA needle	Olympus Smoothshot NA 401D-1521, 21 G
Biopsy forceps	Olympus Endojaw FB-231D
EBUS needle	Olympus Vizishot NA-2015K-4022, 22 G
TTNA needle	HS Hospital Service Chiba needles EC 2115, 21 G
Thoracoscopy (optics and forceps)	Richard Wolf PANOVIEW telescopes, 4 mm, 0°–30°, and rigid optic biopsy forceps, 7 mm

CT, computerized tomography; FDG-PET, fluorodeoxyglucose positron emission tomography; EBUS, endobronchial ultrasound; TBNA, transbronchial needle aspiration; TTNA, transthoracic needle aspiration.

**Table 2:** Clinical features at first diagnosis and at the presentation of the exemplary patients.

	Patient 1	Patient 2	Patient 3	Patient 4
**Age at first diagnosis (years)**	47	48	41	82
**Site of primary tumor**	left leg	back	scalp	unknown
**Breslow**	2.4 mm	7 mm	1.5 mm	NA
**Age at thoracic metastasis (years)**	53	60	52	82
**Site of metastasis**	Lymph nodes	Lung (mass)	Diffuse parenchymal	Pleura
**Diagnostic procedure**	EBUS-TBNA	Fluoroscopy-guided transthoracic needle aspiration	Transbronchial biopsy, BAL	Medical thoracoscopy
**Years between first diagnosis and thoracic metastasis**	6	12	11	0
**Respiratory symptoms**	yes	no	yes	yes
**Molecular profile**	BRAF V600E	BRAF V600E	BRAF V600E	WT
**Survival after thoracic metastasis (months)**	NA	9	4	36
**Other sites of concomitant metastasis**	Abdomen, brain	Brain	Lymph nodes, liver, spleen, bones, kidneys	No

NA, not applicable; WT, wild type; EBUS-TBNA, endobronchial ultrasound transbronchial needle aspiration; BAL, bronchoalveolar lavage.

**Table 3:** Anagraphics and clinical features sorted by pattern of presentation.

	Overall Population	Lymph-Nodes Invasion	Lung Mass(es)	Diffuse Parenchymal Infiltration	Pleural Infiltration	*p*-Value
**Cases (%)**	17 (100)	6 (35)	7 (41)	2 (12)	2 (12)	NA
**Sex (M), *n* (%)**	11 (65)	5 (86)	6 (86)	0 (0)	1 (50)	0.15
**Age at first diagnosis (years), mean** **±** **SD**	57.6 ± 18.7	53.3 ± 15.2	59.6 ± 20.1	39.5 ± 2.1	82 ± 0	0.08
**Age at thoracic metastasis (years), mean** **±** **SD**	64.6 ± 14.2	58 ± 14.2	66.1 ± 13.7	61 ± 12.7	82.5 ± 0.7	0.12
**Years between first diagnosis and thoracic metastasis, mean** **±** **SD**	6.4 ± 8	4 ± 3	9.3 ± 14.1	20.5 ± 13.4	0 ± 0	0.06
**Other sites of metastasis (*n*), mean** **±** **SD**	1.4 ± 1.3	1.5 ± 1	1.3 ± 0.9	2.5 ± 3.5	0.5 ± 0.7	0.81
**Median survival after first diagnosis (months)**	76	130	76	262	31.5	0.06
**Median survival after thoracic metastasis diagnosis (months)**	11	54	8	9	26	0.08
**Breslow (mm), mean** **±** **SD**	3.5 ± 2	2.6 ± 1.9	4.1 ± 1.5	1.5 ± 0	7 ± 0	0.07
**BRAF (%)**	7 (41)	3 (50)	3 (43)	1 (50)	0 (0)	0.64
**Interventional pulmonology diagnosis/patients (diagnostic yield)**	14/16 (87.5)	6/6 (100%)	4/6 (66%)	2/2 (100%)	2/2 (100%)	0.28

SD, standard deviation; NA, not applicable.

**Table 4:** Anagraphics and clinical features sorted by lung parenchymal involvement or nodal/pleural involvement.

	Lung Involvement	Nodal/Pleural Involvement	*p*-Value
**Cases (%)**	9 (53)	8 (47)	NA
**Sex (M), *n* (%)**	6 (67)	5 (63)	>0.9
**Age at first diagnosis (years), mean** **± SD**	55.1 ± 19.6	60.5 ± 18.5	0.57
**Age at thoracic metastasis (years), mean** **± SD**	65 ± 12.9	64.1 ± 16.5	0.9
**Years between first diagnosis and thoracic metastasis, mean** **± SD**	11.8 ± 14	3 ± 3.2	0.1
**Other sites of metastasis (*n*), mean** **± SD**	1.6 ± 1.6	1.2 ± 1	0.64
**Median survival afthe ter first diagnosis (months)**	91	51	0.19
**Median survival after thoracic metastasis diagnosis (months)**	8	39	0.01
**Breslow (mm), mean** **± SD**	3.7 ± 1.7	3.3 ± 1.4	0.49
**BRAF (%)**	4 (44)	3 (38)	>0.9
**Interventional pulmonology diagnosis/patients (diagnostic yield)**	6/8 (75)	8/8 (100)	0.47

SD, standard deviation; NA not applicable.

## Data Availability

The data presented in this study are available on request from the corresponding author. The data are not publicly available due to the privacy policy for clinical information applied in Italy.
